# Supplementary material for: Acquired CRISPR spacers and rhamnose-glucose polysaccharide defects confer resistance to Streptococcus mutans phage ɸAPCM01
Source: bioRxiv. 2025 May 5:2025.05.05.652303. Preprint. [Version 1] doi: 10.1101/2025.05.05.652303 (PMC12247853; doi:10.1101/2025.05.05.652303)
Supplement: 1 [file NIHPP2025.05.05.652303v1-supplement-1.pdf]

bioRxiv preprint doi: https://doi.org/10.1101/2025.05.05.652303; this version posted May 7, 2025. The copyright holder for this preprint (which was not certified by peer review) is the author/funder, who has granted bioRxiv a license to display the preprint in perpetuity. It is made available under aCC-BY 4.0 International license.

Supplemental Material

Title: Acquired CRISPR spacers and rhamnose-glucose polysaccharide defects confer resistance to *Streptococcus mutans* phage ϕAPCM01

Lucas A. Wall and Daniel Wall

Department of Molecular Biology, University of Wyoming, Laramie, WY 82071, USA

bioRxiv preprint doi: https://doi.org/10.1101/2025.05.05.652303; this version posted May 7, 2025. The copyright holder for this preprint (which was not certified by peer review) is the author/funder, who has granted bioRxiv a license to display the preprint in perpetuity. It is made available under aCC-BY 4.0 International license.

Table S1 Endogenous CRISPR spacers in host DPC6143

Spacer	Spacer sequence	Phage/species hit	Identity
1	AAAATGCTAGTTGAAACA TTTTCTAGTTTT	Phage M102	28/30 bp
2	CGATATTGGAACAACTGT TACAACTCCAAC	*Bacillus cytoxillus* strain E28	28/30 bp
3	TGGAACTGTCCAGAATTG CAATAGCAGCAC	No relevant hit	
4	TGTCTATCTAAAAAAAGA TAATGGTGAAAA	No relevant hit	
5	GTAAAAAACGGAGAGTTG TATGCATATTTT	No relevant hit	
6	TCCGAGAATAGGAGCTT GACAGTTCCCGTC	No relevant hit	
7	AAATTTCTTATTAAGAAAA GTGAGATGAAA	No relevant hit	
8	CAGCAAGAACGCAGTAG TTTATGTAGGGGG	Phage ϕAPCM01	27/30 bp
9	TAGACGCAGAACTATTAG TTTTGAGGATAC	No relevant hit	
10	CGCTAGTAATTAATGGTA AACAGCATAATA	*Streptococcus* phage phiKSM96	23/30 bp
11	TAATGAGAAAGTCATAGT CCATGTCTACCG	No relevant hit	
12	GAGAAAGCAGAAAATACT TCTATTGGTTCT	*Streptococcus* phage phiKSM96<br>*S. mutans* KCOM 1054 (spacer)	29/30 bp<br>30/30 bp
13	GCCGTCAGAGTTCTTCCA CTCTTGCTCT	Phage *Caudoviricetes* sp. ctNo011	30/30 bp
14	CTCCAATTGAAAATATTG ATCCAGGTTATT	Phage *Caudoviricetes* sp. ctNo011<br>*S. mutans* KCOM 1054 (spacer)	28/30 bp<br>30/30 bp
15	AGCGAAATTGGAAGATGT TTAGTGTTGCTT	No relevant hit	
16	ATAGTACGAAAAATGAAA ATTTAGGAGTTC	*S. mutans* UA159 and *S. mutans* KCOM 1054 (spacers)	30/30 bp
17	TTGCTAAAAAAGGTGATG TGGTTTATAAGT	*Candidatus Pelagibacter giovannonii* NP1	27/30 bp
18	AGCTATTAAACGTGGTTT AACACAATCTTG	No relevant hits	
19	CTATTGCTGTCTTGTAGT GCTTCGAGTCAA	Phage *Caudoviricetes* sp. ctQS92	24/30 bp
20	TACTAAGCAGTTTGCAGA TGAAGACCCGCG	Phages M102 & M102AD	27/30 bp
21	CCTTTTGACCTGACTAAC CAGGAGCAAGAG	*S. mutans* phage smHBZ8	27/30 bp
22	TAATTCGTCAAATTCTCC ACCCAGAAGCG	Phage M102	28/29 bp
23	ACCGCGTTGTTCTAAAAA TCTAATTTGTTT	*S. mutans* KCOM 1054 (spacer)	29/30 bp
24	TGGACGCAAGAATATCTA TTTAAATGGTTC	No relevant hits	
25	AAATCCACCAACTGACTC AATTGATCCATT	No relevant hits	

bioRxiv preprint doi: https://doi.org/10.1101/2025.05.05.652303; this version posted May 7, 2025. The copyright holder for this preprint (which was not certified by peer review) is the author/funder, who has granted bioRxiv a license to display the preprint in perpetuity. It is made available under aCC-BY 4.0 International license.

26	CCAAAATTTTTAGCTCAA AAATACGGCAAA	Phages ϕAPCM01 & smHBZ8<br>Phage M102AD	28/30 bp<br>23/30 bp
27	AAAGCTGATTTTTTTATTT TTCCAGCCAGT	*Streptococcus dysgalactiae* subsp. *equisimilis* strain MGGS36030	30/30 bp
28	AATAGAATCAAATTCTCC TAAGTCAGTTAA	*S. mutans* and *Streptococcus* spp.	30/30 bp

bioRxiv preprint doi: https://doi.org/10.1101/2025.05.05.652303; this version posted May 7, 2025. The copyright holder for this preprint (which was not certified by peer review) is the author/funder, who has granted bioRxiv a license to display the preprint in perpetuity. It is made available under aCC-BY 4.0 International license.

Table S2 Mutations in ϕAPCM01 resistant isolates

Strains	Protein/element	Mutations	RefSeq	DNA coordinates
DPC6143 (WT)	N/A	N/A	N/A	
$rgpX^{W190NS-CR1}$	RgpX<br>CRISPR spacer<br>ZupT<br>*serP2-I-rqcH*	W190NS<br>CRISPR 1<br>M48I<br>Intergenic G->T	WP_002301943.1<br>N/A<br>WP_002262376.1<br>N/A	1,246,166<br>753136-753166<br>1,986,979<br>702,516
$rgpX^{P521H-CR2ab}$	RgpX<br>CRISPR spacer<br>CRISPR spacer	P521H<br>CRISPR 2a<br>CRISPR 2b	WP_002301943.1<br>N/A<br>N/A	1,245,173<br>753136-753166<br>753992-754027
$rgpF^{E491K}$	RgpF<br>Glycoside hydrolase<br>Hypo peptidase	E491K<br>G249R<br>A166S	WP_002261980.1<br>WP_002263319.1<br>WP_019803208.1	1,250,491<br>1,365,248<br>1,525,118
$rgpF^{T419P-CR3ab}$	RgpF<br>CRISPR spacer<br>CRISPR spacer<br>Glycoside hydrolase	T419P<br>CRISPR 3a<br>CRISPR 3b<br>G249R	WP_002261980.1<br>N/A<br>N/A<br>WP_002263319.1	1,250,707<br>753136-753166<br>754322-754357<br>1,365,248
$rgpX^{FS}$	RgpX<br>Hypo zinc-binding alcohol dehydrogenase	ΔG codon 194<br>+A insertion	WP_002301943.1<br>WP_019802715.1	1,246,154<br>994,472
$rgpF^{G382S-A}$	RgpF<br>Glycoside hydrolase	G382S<br>G249R	WP_002261980.1<br>WP_002263319.1	1,250,818<br>1,365,248
$rgpF^{G382S-B}$	RgpF<br>Glycoside hydrolase<br>tRNA<br>tRNA<br>23S ribosomal RNA<br>16S ribosomal RNA	G382S<br>G249R<br>T->G<br>Deletion<br>Multiple mutations<br>Multiple mutations	WP_002261980.1<br>WP_002263319.1<br>N/A<br>N/A<br>N/AN/A	1,250,818<br>1,365,248<br>22,362<br>22,386<br>1,862,014-1,863,946<br>1,864,804-1,865,996
$rgpD^{F162Y}$	RgpD	F162Y	WP_002267627.1	1,254,111
CR4	CRISPR spacer	CRISPR 4	N/A	753135-753164
CR5abc	CRISPR spacer<br>CRISPR spacer<br>CRISPR spacer	CRISPR 5a<br>CRISPR 5b<br>CRISPR 5c	N/A<br>N/A<br>N/A	753172-753268<br>753701-753928<br>754520-754555
CR6ab	CRISPR spacer<br>CRISPR spacer<br>Glycoside hydrolase	CRISPR 6a<br>CRISPR 6b<br>G249R	N/A<br>N/A<br>WP_002263319.1	753136-754555<br>754585-754620<br>1,365,248
CR7	CRISPR spacer<br>Hypo peptidase-I-ccpA	CRISPR 7<br>G->T	N/A<br>N/A	753134-753171<br>546,203
CR8ab	CRISPR spacer<br>CRISPR spacer	CRISPR 8a<br>CRISPR 8b	N/A<br>N/A	753136-753766<br>754520-754620
CR9abc	CRISPR spacer<br>CRISPR spacer<br>CRISPR spacer<br>Hypo<br>Glycoside hydrolase	CRISPR 9a<br>CRISPR 9b<br>CRISPR 9c<br>L122R<br>G249R	N/A<br>N/A<br>N/A<br>WP_002273056.1<br>WP_002263319.1	753136-753171<br>753136-753171<br>753991-754028<br>582,063<br>1,365,248
CR10	CRISPR spacer	CRISPR 10	N/A	753135-754159

Hypo, hypothetical; N/A, not applicable

bioRxiv preprint doi: https://doi.org/10.1101/2025.05.05.652303; this version posted May 7, 2025. The copyright holder for this preprint (which was not certified by peer review) is the author/funder, who has granted bioRxiv a license to display the preprint in perpetuity. It is made available under aCC-BY 4.0 International license.

Table S3 DNA sequence of acquired spacers and ϕAPCM01 targets

Strain	CRISPR ID	bp	Sequence	Phage gene product
rgpX$^{W190NS\text{-}CR1}$	CRISPR 1	30	TTGCTAAGAACGACGGCACAGCAAGCGCTA	Minor tail protein
rgpX$^{P521H\text{-}CR2ab}$	CRISPR 2a<br>CRISPR 2b	30<br>30	GAAACACAGAGCTAGTCCGCAAGAGCTACA<br>GTTTACGCTGTAATCATTTTGATTGTAATT	RecT-like-ssDNA protein<br>Portal Protein
rgpF$^{T419P\text{-}CR3ab}$	CRISPR 3a<br>CRISPR 3b	30<br>30	AACTATGCAGTAACTCCCGACCCCGTGTGG<br>GCAATAATGGAAGTTGCTGGTGAAATGACC	Endolysin<br>Tape measure protein
CR4	CRISPR 4	29	CGCCGTTGATATTAATAGTCATTGTTGCG	Phage structural protein
CR5abc	CRISPR 5a<br>CRISPR 5b<br>CRISPR 5c	29<br>28<br>30	CACGTTTCCTAAAATGAAACCTGCAATAA<br>CCGTTTACGGTCATCCTTTCGGACACAC<br>GCCGTATACAGTATCAAGTGCATAGACAGT	Tape measure protein<br>Endolysin<br>Terminase large subunit
CR6ab	CRISPR 6a<br>CRISPR 6b	30<br>30	TGTTGAGCTGAGCCAGGAGTTACTCCGCCG<br>GAAACACACAAAGCAAAAACGAAAGCAAAC	Major tail protein<br>Major tail protein
CR7	CRISPR7	28	TGTAAGTCTCTGATGTCAATCTCATCTA	Hypothetical protein
CR8ab	CRISPR 8a<br>CRISPR 8b	29<br>30	GCGGGCTCAACTTGATCCGTGGCGCACTT<br>ATCAGCTCCACAAATCAGCTTTAATTTTAA	Major tail protein<br>Tape measure protein
CR9abc	CRISPR 9a<br>CRISPR 9b<br>CRISPR 9c	30<br>30<br>28	GACTATATCTTTTTCTTTGTGTTTAATTAA<br>TCTGGTTTTGAACCAATAAATTTATAAGGC<br>TGGTTTAGCCATGATTTCTCCTCTTTTC	Distal tail protein<br>Intergenic<br>Hypothetical protein
CR10	CRISPR 10	29	CAATCTCTAAGCGTTGGTGCTCAATGGAA	Capsid protein

bioRxiv preprint doi: https://doi.org/10.1101/2025.05.05.652303; this version posted May 7, 2025. The copyright holder for this preprint (which was not certified by peer review) is the author/funder, who has granted bioRxiv a license to display the preprint in perpetuity. It is made available under aCC-BY 4.0 International license.

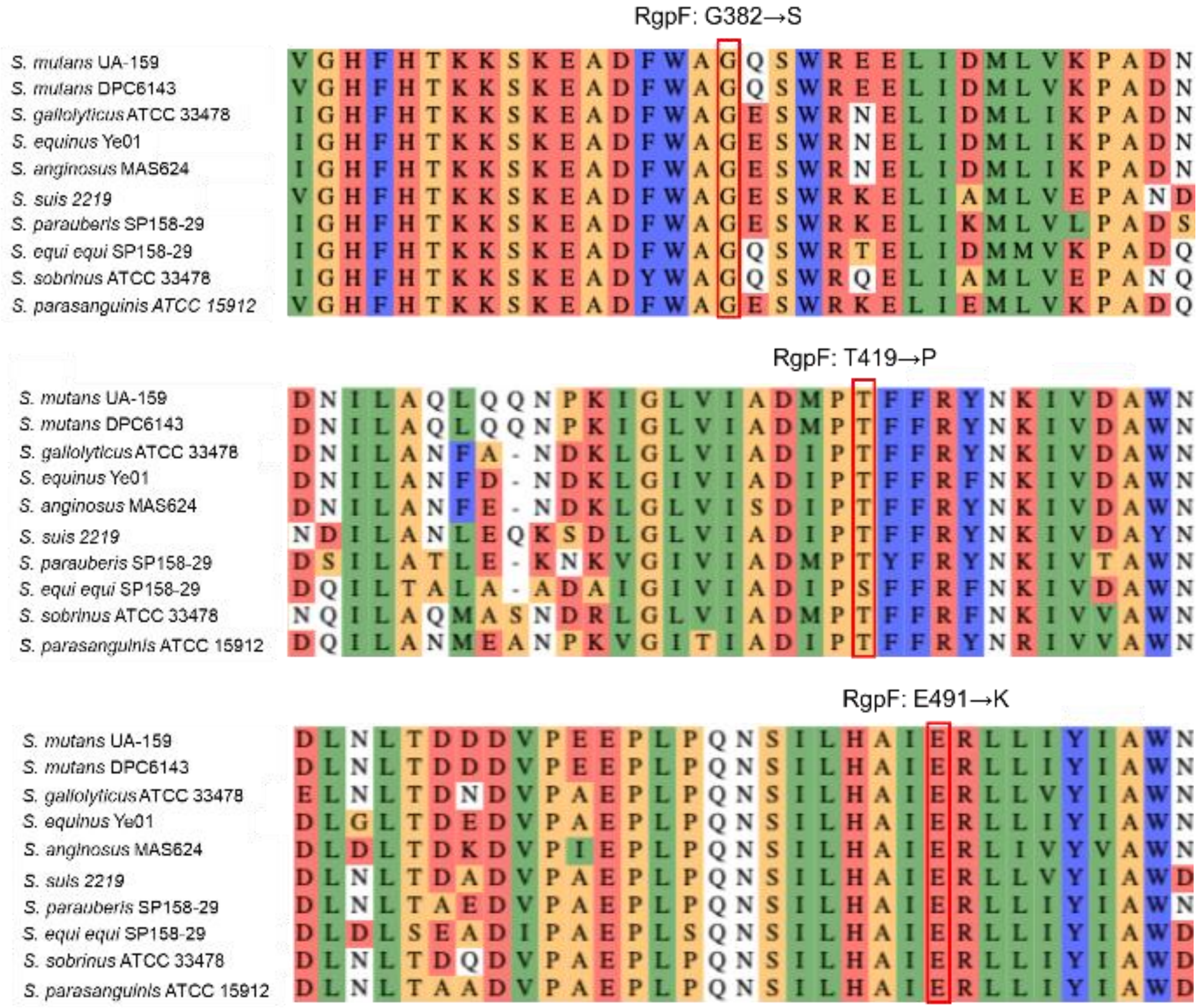

Figure S1. Sequence conservation among *Streptococcus* species around *S. mutans* RgpF substituted residues that confer ϕAPCM01 resistance.

bioRxiv preprint doi: https://doi.org/10.1101/2025.05.05.652303; this version posted May 7, 2025. The copyright holder for this preprint (which was not certified by peer review) is the author/funder, who has granted bioRxiv a license to display the preprint in perpetuity. It is made available under aCC-BY 4.0 International license.

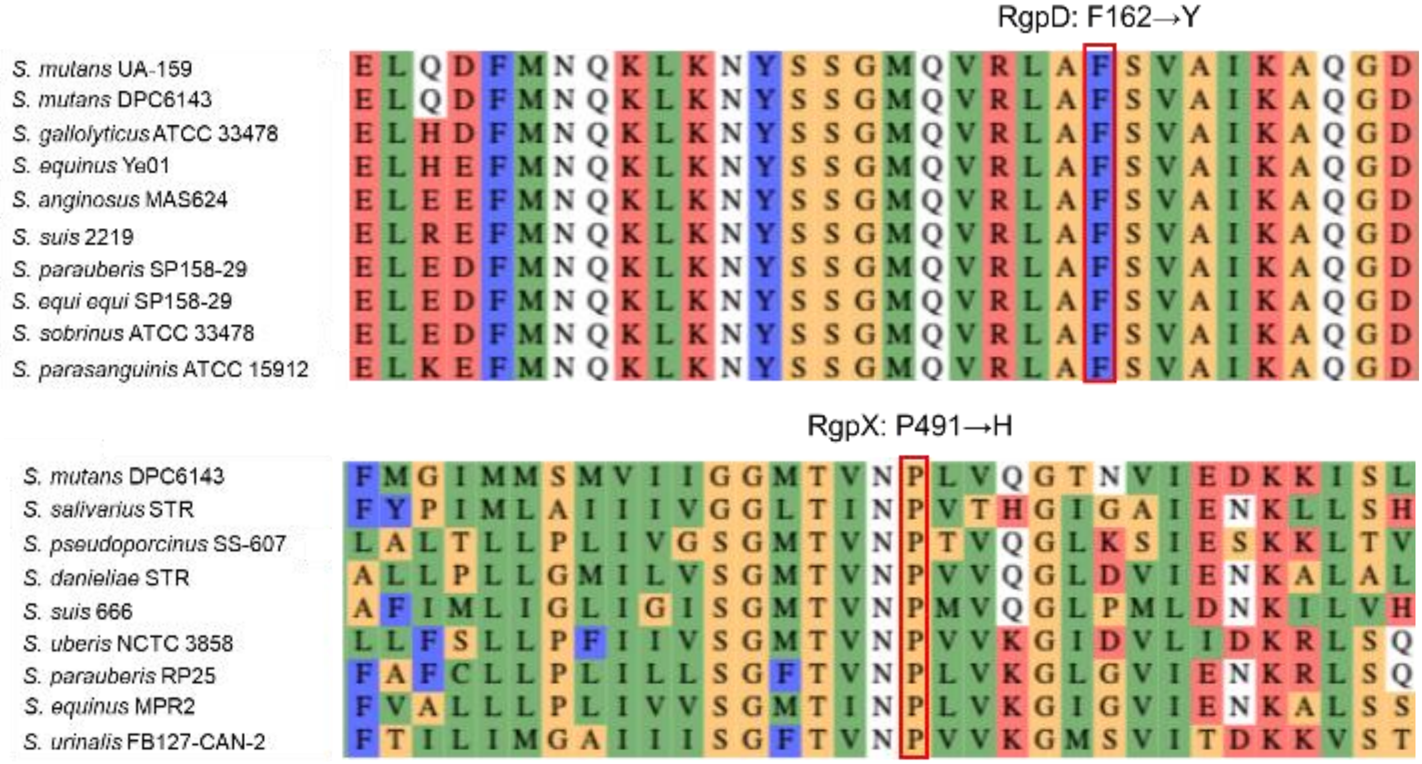

Figure S2. Sequence conservation among *Streptococcus* species around *S. mutans* RgpD and RgpX substituted residues that confer ϕAPCM01 resistance.

bioRxiv preprint doi: https://doi.org/10.1101/2025.05.05.652303; this version posted May 7, 2025. The copyright holder for this preprint (which was not certified by peer review) is the author/funder, who has granted bioRxiv a license to display the preprint in perpetuity. It is made available under aCC-BY 4.0 International license.

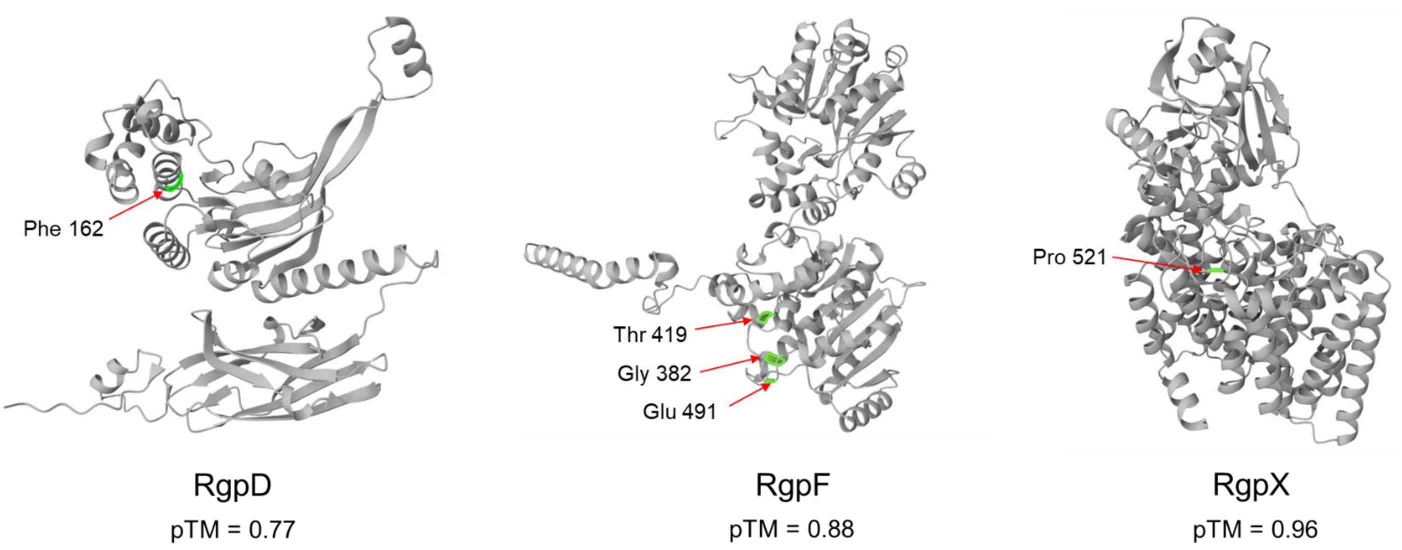

Figure S3. AlphaFold 3 predicted structures of Rgp proteins. Residues with substitutions that confer ϕAPCM01 resistance highlighted. All pTM scores indicate high confidence structures.

bioRxiv preprint doi: https://doi.org/10.1101/2025.05.05.652303; this version posted May 7, 2025. The copyright holder for this preprint (which was not certified by peer review) is the author/funder, who has granted bioRxiv a license to display the preprint in perpetuity. It is made available under aCC-BY 4.0 International license.

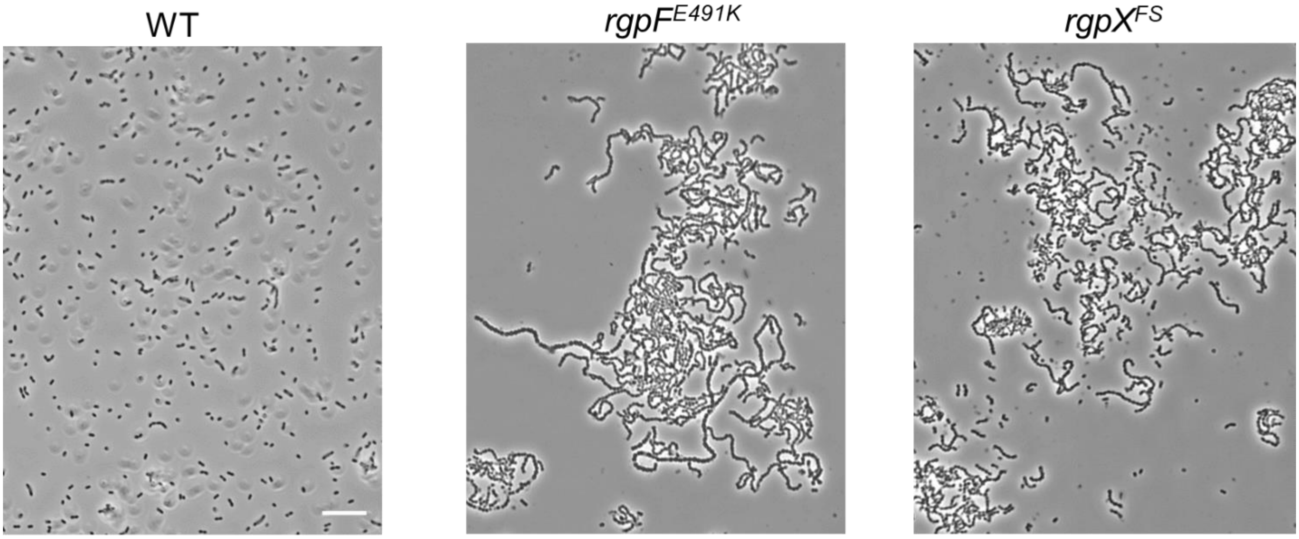

Figure S4. Culture tube samples from sediment shown in Figure 6A. Scale bar, 10 μM.
